# Incorporating exon–exon junction reads enhances differential splicing detection

**DOI:** 10.1186/s12859-025-06210-4

**Published:** 2025-07-24

**Authors:** Mai T. Pham, Michael J. G. Milevskiy, Jane E. Visvader, Yunshun Chen

**Affiliations:** 1 ACRF Cancer Biology and Stem Cells Division, The Walter and Eliza Hall Institute of Medical Research, 1G Royal Parade, Parkville, VIC 3052 Australia; 2 Bioinformatics Division, The Walter and Eliza Hall Institute of Medical Research, 1G Royal Parade, Parkville, VIC 3052 Australia; 3 Department of Medical Biology, The University of Melbourne, Parkville, VIC 3010 Australia

**Keywords:** Exon junction, Alternative splicing, RNA-sequencing

## Abstract

**Background:**

RNA sequencing (RNA-seq) is a gold standard technology for studying gene and transcript expression. Different transcripts from the same gene are usually determined by varying combinations of exons within the gene, formed by splicing events. One method of studying differential alternative splicing between groups in short-read RNA-seq experiments is through differential exon usage (DEU) analysis, which uses exon-level read counts along with downstream statistical testing strategies. However, the standard exon counting method does not consider exon-junction information, which may reduce the statistical power in detecting splicing alterations.

**Results:**

We present a new workflow for differential splicing analysis, called differential exon-junction usage (DEJU). This DEJU analysis workflow adopts a new feature quantification approach that jointly summarises exon and exon–exon junction reads, which are then integrated into the established *Rsubread-edgeR/limma* frameworks. We performed comprehensive simulation studies to benchmark the performance of DEJU against existing methods. We also applied DEJU to a mouse mammary gland RNA-seq dataset, revealing biologically meaningful splicing events that could not be detected previously.

**Conclusions:**

We demonstrate that incorporating exon–exon junction reads significantly improves the detection of differential splicing events. The proposed DEJU workflow offers increased statistical power and computational efficiency compared to widely used existing approaches, while effectively controlling the false discovery rate.

## Background

Alternative splicing (AS) is a pivotal post-transcriptional regulatory mechanism in eukaryotes that enables the generation of multiple mRNA isoforms by selectively including or excluding exonic and intronic regions, or by utilizing alternative splice sites [1]. This process contributes to the transcriptomic and proteomic diversity, allowing cells to adapt to functional demands for development and differentiation [2]. In the human genome, dysregulated splicing has been extensively linked to human diseases, notably cancer, which is associated with the expression of mis-spliced isoforms of cancer-related genes leading to cellular transformation, proliferation, metastasis, and therapeutic response [3–5]. Identification of such splicing alterations is crucial for the development of diagnostic and therapeutic targets, some of which have been used clinically in cancer treatment [6].

Over the past decade, bulk RNA-sequencing (RNA-seq) has emerged as a powerful and cost-effective technology for measuring the expression levels of genomic features such as genes, transcripts, and exons, based on quantified RNA-seq reads mapped to these features [7, 8]. Exon-level read counts can then be used for differential exon usage (DEU) analysis [9], enabling the detection of differential splicing (DS) between two groups of conditions, indicative of splicing alterations. This approach enables the rapid identification of genes that may be differentially spliced, referred to hereafter as DEU genes, without requiring the specification of the exact isoforms present in different groups.

There are commonly used R/Bioconductor packages for DEU analysis, including *DEXSeq* [9], *JunctionSeq* [10], *diffSplice* function in *limma* [11], and *diffSpliceDGE* function in *edgeR* [12]. *edgeR* and *limma* have currently been included functionality to perform DEU analysis based on exon-level read quantification by *featureCounts* function in the *Rsubread* package [13]. In the current approach, exon reads mapped to regions spanning two exons, known as exon–exon junction reads, will, by default, contribute one count to both exons, leading to a double-counting issue. Moreover, this current approach was originally developed to identify differential usage of flattened and merged exons that is limited in detecting exon extension or truncation involving alternative splice sites, retained introns, or nested exon skipping. This limitation arises from the lack of information regarding junction sites. On the other hand, *DEXSeq* detects DS signals by segmenting exons at alternative splice sites into smaller exonic bins and applying another statistical method to detect DEU genes. *JunctionSeq*, an extension of *DEXSeq*, performs exon–exon junction read counting, enabling differential exon and junction usage analysis, which achieves more DS signals compared to the existing DEU approach [14]. This indicates the advantage of incorporating junction read counts into DS analysis.

To address the double-counting problem of exon-level read counts and enhance the overall performance of DEU analysis within the *Rsubread-edgeR-limma* framework, we propose a differential exon-junction usage (DEJU) analysis workflow. This workflow incorporates exon-junction reads into the existing DEU analysis process. Specifically, we utilize a splice-aware aligner *STAR* [15] to identify splice junctions and subsequently quantify junction reads using the *featureCounts* function with settings that differentiate internal exon reads from exon-junction reads. In the final read count matrix, exon-junctions are included as features alongside exons. This approach ensures that each exon-junction read is uniquely assigned to a single feature, thereby effectively resolving the double-counting issue and ensuring that the resulting library sizes accurately reflect the true number of sequence reads.

In this article, we benchmark the performance of our proposed DEJU workflow (*DEJU-edgeR* and *DEJU-limma*), which integrates both exon and exon–exon junction information, against the current DEU approaches that do not consider junctions (*DEU-edgeR* and *DEU-limma*), as well as other popular methods such as *DEXseq* and *JunctionSeq*. We use simulated bulk RNA-seq datasets with various designed splicing patterns, where the ground truths are known, to evaluate the accuracy and effectiveness of these methods. The DEJU workflow is also evaluated for its robustness in terms of precision and sensitivity in detecting DEU genes across various combinations of library sizes and sample sizes. We show that our DEJU analysis workflow is more precise, powerful, and flexible in detecting a broader range of splicing events compared to existing popular methods. It enhances statistical power while effectively controlling the false discovery rate (FDR). The DEJU analysis process is also efficient in time and memory usage. Additionally, this workflow is tested on a mouse bulk RNA-seq experiment involving two mammary epithelial cell (MEC) types (luminal progenitor and mature luminal) [16], revealing biologically meaningful findings related to AS events that could not be detected previously.

## Results

### Differential exon-junction usage (DEJU) analysis workflow

**Fig. 1:**
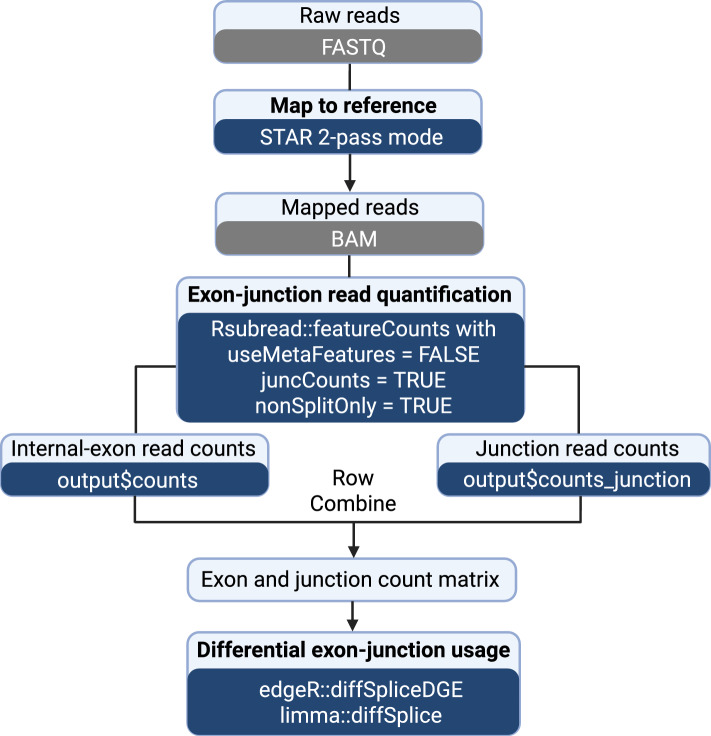
The complete DEJU analysis workflow under the Rsubread-edgeR-limma framework

We propose a complete workflow for differential splicing analysis of short-read bulk RNA-seq experiments, called differential exon-junction usage (DEJU). This DEJU workflow utilizes the *STAR* and *Rsubread* for read alignment and feature quantification respectively, and it leverages the *edgeR* or *limma* packages for downstream statistical analysis (Fig. 1). Details of key workflow steps are described below.

RNA-seq reads are first aligned to the reference genome using *STAR* aligner using the 2-pass mapping mode with re-generated genome. To achieve the highest sensitivity to novel junction detection, a list of junctions detected by the 1-pass mapping from all samples across groups of conditions are collapsed and filtered. The resulting set of junctions are subsequently used to re-index the reference genome for the second round of mapping. Junction reads aligned to junctions that pass a certain filtering threshold (e.g., more than three uniquely mapping reads across all samples), are kept in the alignment BAM files using the --outFilterType BySJout option.

The aligned reads are then quantified by the *featureCounts* function in *Rsubread* using the flattened and merged exon annotation with the argument useMetaFeatures=FALSE. The nonSplitOnly argument in *featureCounts* allows quantification of internal exon read counts, while the juncCounts argument enables *featureCounts* to return an extra count matrix for exon–exon junctions. By default, both nonSplitOnly and juncCounts are set to FALSE. Under this DEJU workflow, both arguments are set to TRUE to obtain both internal exon and junction count matrices simultaneously. The reference genome-generated junction database is subsequently incorporated into our DEJU workflow to improve the assignment of annotated junctions to genes. Internal exon and processed junction count matrices are then concatenated into a single exon-junction count matrix. Exons and junctions with low number of mapped reads are filtered by the *filterByExpr* function in *edgeR*. The trimmed mean of M values (TMM) normalization [17] is performed using the *normLibSizes* function in the *edgeR* package to account for the composition biases between libraries.

The downstream differential exon-junction usage analyses can be performed using either the *diffSpliceDGE* function in *edgeR* or the *diffSplice* function in *limma*. These two functions identify features (both exons and junctions) that are differentially used between the groups. The feature-level test results are then summarized at the gene level using either the Simes method [18], which combines feature-level *P* values within each gene, or an *F*-test, which combines feature-level quasi-likelihood *F*-test statistics for that gene [12].

### False discovery rate and statistical power

We evaluated the performance of various DEU and DEJU workflows with respect to their power in detecting DEU genes featuring different AS patterns, including exon skipping (ES), mutually exclusive exon (MXE), alternative 3′/5′ splice site (ASS), and intron retention (IR), and their ability to control FDR. See Supplementary Table S1, Additional File 2 for full results. Table 1 showed the statistical power and FDR of *DEJU-edgeR* and *DEJU-limma* on simulated datasets under different simulation settings and for different splicing patterns. In general, the ability to detect DEU genes increased with larger sample sizes, particularly evident in the improved detection of ASS and IR events. *DEJU-edgeR* effectively controlled FDR at the nominal rate of 0.05 for all splicing events, although it was slightly more conservative compared to *DEJU-limma*. In contrast, *DEJU-limma* struggled to control the FDR as effectively as *DEJU-edgeR*, particularly in detecting MXE events.

**Table 1:** FDR and statistical power in the DEU detection of *DEJU-edgeR* and *DEJU-limma* using the gene-level Simes method under different sample sizes (n)

Pattern	n	DEJU-edgeR		DEJU-limma	
		FDR	Power	FDR	Power
ES	3	0.022	0.977	0.043	0.975
	5	0.029	0.991	0.044	0.990
	10	0.038	0.992	0.051	0.992
MXE	3	0.030	0.990	0.061	0.991
	5	0.040	0.993	0.062	0.995
	10	0.045	0.995	0.063	0.995
ASS	3	0.027	0.839	0.038	0.877
	5	0.027	0.927	0.037	0.947
	10	0.038	0.977	0.047	0.979
IR	3	0.030	0.866	0.042	0.880
	5	0.031	0.934	0.041	0.940
	10	0.042	0.964	0.050	0.968

The table shows the FDR and power in DEU detection under four splicing patterns, with balanced library sizes and 250 genuine DEU genes for each splicing pattern. Two transcripts are simulated for each gene. Results are averaged over 20 independent simulation runs for each sample size

**Fig. 2:**
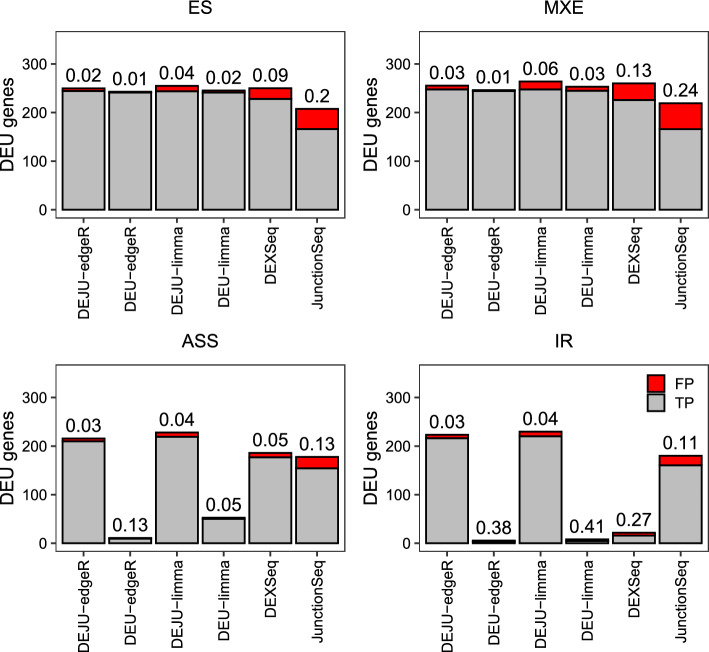
FDR of the benchmarked workflows in the DEU analysis with 4 different splicing pattern scenarios—ES, MXE, ASS, and IR. Two transcripts are simulated for each gene. Stacked barplots showing the average number of true (gray) and false (red) positive DEU genes at nominal 5% FDR. The observed FDR is shown over each bar. Results are averaged over 20 independent simulation runs with 75 bp paired-end reads and 250 genuine DEU genes simulated for each splicing pattern. DEU/DEJU analysis results are shown using the gene-level Simes method

Figure 2 showed the observed number of true positive and false positive DEU genes for benchmarked methods, including *DEJU-edgeR*, *DEJU-limma*, *DEU-edgeR*, *DEU-limma*, *DEXSeq*, and *JunctionSeq*, under a nominal FDR control of 0.05, in a simulation scenario with three samples per group and balanced library sizes. Full details on FDR control across other simulation scenarios are provided in Supplementary Fig. S1, Additional File 1. Overall, the DEJU method implemented in *edgeR* and *limma* demonstrated superior FDR control at 0.05 compared to other methods, particularly in detecting ASS and RI events. *DEXSeq* detected a substantially higher number of true positive ASS cases than *DEU-edgeR* and *DEU-limma*. Notably, IR events were detectable exclusively by DEJU-based workflows (*DEJU-edgeR/limma* and *JunctionSeq*), highlighting the advantage of incorporating junction reads into DS detection. However, *JunctionSeq* was less effective in controlling FDR in discovering such cases compared to our DEJU method. We also benchmarked the edgeR and limma workflows using only junction counts, denoted as *junc-edgeR* and *junc-limma*. These junction-only approaches effectively controlled the FDR below 0.05 and outperformed DEU methods that do not incorporate junction information (Supplementary Fig. S1, Additional File 1).

**Fig. 3:**
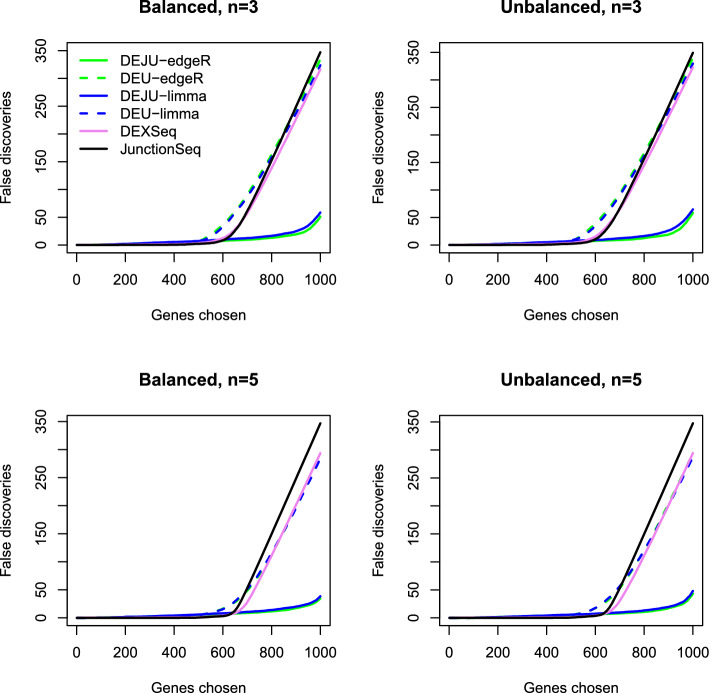
Panels show the average number of false discoveries as a function of the number of chosen DEU genes across the combination of balanced/unbalanced library size and sample sizes (3,5 replicates per group). Two transcripts are simulated for each gene. Results are averaged over 20 independent simulation runs with 75 bp paired-end reads. DEU/DEJU analysis results are shown using the gene-level Simes method

To further evaluate methods for FDR control, we assessed the number of false discoveries in the set of top-ranked most significant DEU genes identified by each method (Fig. 3). Overall, *DEJU-edgeR* and *DEJU-limma* consistently produced the smallest number of false discoveries among all methods for any number of top-ranked DEU genes. Across various library and sample size configurations, *DEU-edgeR*, *DEU-limma*, *DEXSeq*, and *JunctionSeq* exhibited more false positive DEU genes for any given number of top-ranked DEU genes. In our simulations of 250 cases per splicing event, ES and MXE were readily detected by all tools, whereas ASS and RI were more challenging to identify, except for *DEJU-edgeR/limma* and *junc-edgeR/limma*, which effectively captured these complex splicing events with consistently low FDR (Fig. 2, Supplementary Figures S1–S2, Additional File 1). Therefore, the number of false discoveries of other methods increased significantly beyond the top-ranked 500–600 DEU genes (Fig. 3, Supplementary Figures S3 and S4, Additional File 1). However, increasing the number of replicates per group led to a slight reduction in false discoveries for most benchmarked methods. Compared to the gene-level Simes method, DEU and DEJU analyses of *edgeR* and *limma* using the gene-level *F*-test yielded slightly higher numbers of false discoveries. Nevertheless, both tests produced comparable results in scenarios with 10 replicates per group (Supplementary Figs. S3 and S4, Additional File 1).

*DEJU-edgeR* and *DEJU-limma* consistently maintained an empirical FDR below the nominal threshold of 0.05 and demonstrated substantially higher power and flexibility in detecting various splicing patterns from the transcriptome with two or five transcripts per gene, regardless of replicate number or library size (Supplementary Figs. S1–S7, Additional File 1). Notably, in the more complex five-transcript-per-gene scenario, both workflows continued to control FDR effectively, though sensitivity declined somewhat, particularly for ASS and RI events. However, increasing the number of replicates helped preserve detection power.

Overall, these results underscore the superior performance of the DEJU workflow in *edgeR* and *limma*, achieving levels of accuracy and FDR control that surpass existing DEU methods. While junction-only approaches *junc-edgeR/limma* also maintained proper FDR control, *DEJU-edgeR/limma* provided greater statistical power and more accurate prioritization of top-ranked genes. Notably, their performance further improves with larger sample sizes.

### Computational resources

We compared the computational speed and resources required for the downstream statistical testing part under different workflows. Both *DEU-edgeR* and *DEJU-edgeR* use the *diffSpliceDGE* function for statistical analysis, whereas both *DEU-limma* and *DEJU-limma* use the *diffSplice*. Table 2 summarises runtime and memory cost for each method in analyzing RNA-seq reads with the balanced library sizes across samples, benchmarked in both parallel and serial modes to illustrate work-load distribution and method efficiency. Based on these metrics, we observed that both *diffSpliceDGE* and *diffSplice* functions, regardless of DEU or DEJU workflows, had substantially lower compute cost and turnaround time, while consistently maintaining their accuracy and power in detecting DEU genes with different splicing patterns. On the other hand, the computational time and resources of *DEXSeq* and *JunctionSeq* scaled up proportionally with increasing sample sizes. The consumption in computing resources of these methods for unbalanced library sizes is shown in Supplementary Table S2, Additional File 1. These results demonstrate the robustness and computational efficiency of *edgeR diffSpliceDGE* and *limma diffSplice* for DEU/DEJU analysis, even as the number of replicate samples increases.

**Table 2:** Compute cost of compared DEU detection tools with increasing sample sizes (n)

n	Method	Walltime (min)		Memory (Gb)	
		Parallel	Serial	Parallel	Serial
3	EdgeR	–	0.47	–	0.64
	Limma	–	0.33	–	0.55
	DEXSeq	9.74	18.29	4.81	2.71
	JunctionSeq	10.12	16.06	6.87	3.11
5	EdgeR	–	0.64	–	0.63
	limma	–	0.42	–	0.63
	DEXSeq	11.56	32.85	4.91	3.37
	JunctionSeq	10.57	24.54	8.61	4.24
10	EdgeR	–	0.71	–	0.80
	limma	–	0.41	–	0.75
	DEXSeq	29.15	92.55	6.27	4.99
	JunctionSeq	20.55	58.39	11.36	5.34

All tests are carried out on Intel Xeon E5-2690 v4 server with data on local drive. Parallel tests use 4 cores, serial tests use 1 core. Memory columns show peak RSS. Walltime and memory usage of the *diffSpliceDGE* function of *edgeR* and the *diffSplice* function of *limma* is for both DEU and DEJU analyses. No parallelization is available for these methods

### Differential exon-junction usage analysis for mouse mammary epithelial cell lines

Here, we detected DEU genes between luminal progenitor (LP) and mature luminal (ML) MECs from the Illumina short paired-end read RNA-seq experiments with three biological replicate samples per cell line. Libraries were sequenced with an Illumina NextSeq 500 sequencing system, generating 34–82 millions of 80–81 bp read-pairs per sample. DEU genes were identified using our DEJU analysis workflow, and the library sizes of internal exon counts, junction counts, and exon-junction counts of *DEU-edgeR* and *DEJU-edgeR* were recorded (Supplementary Table S3, Additional File 1). Overall, the proposed DEJU workflow gave library sizes more precisely 37–86 million reads—as the actual sequencing libraries compared to the existing DEU analysis workflow. Diagnostic plots of the exon-junction read quantification of DEU/DEJU analysis workflow implemented in *edgeR* are shown in Supplementary Fig. S8, Additional File 1.

**Fig. 4:**
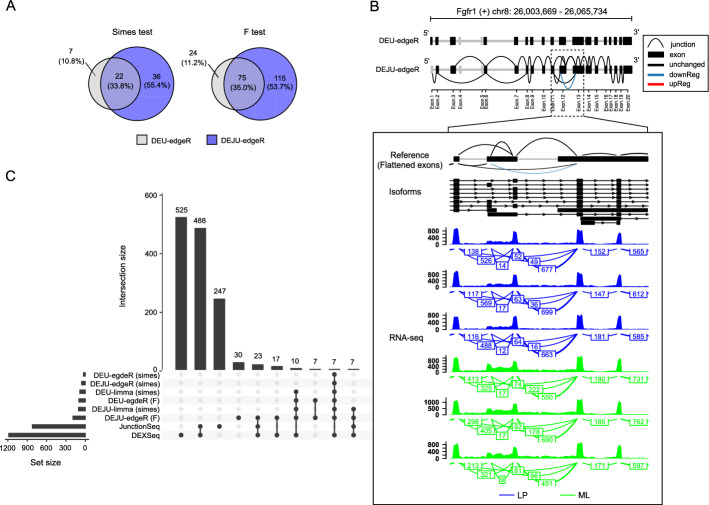
Panels (**A**–**C**) shows DEU detection results between LP and ML samples. **A** VennDiagram shows the number of DEU gene detections by *DEU-edgeR* (gray) and *DEJU-edgeR* (purple); **B** An example of a DEU gene, *Fgfr1*, with a down-regulated junction in LP samples exclusively detected by *DEJU-edgeR* with the gene-level Simes method (adjusted *P* value = 0.018) compared to *DEU-edgeR* (adjusted *P* value = 1). (Top panels) Schematic exon-junction plots showing the up-regulated (red), down-regulated (blue), and non-differentially (black) used exons and junctions in LP against ML samples; (Middle panels) a UCSC comprehensive transcript annotation track of mm39; (Bottom panels) Sashimi plots alongside the coverage of RNA-seq reads of LP (blue) and ML (green) samples. Exon–exon junction read counts are shown in a box; **C** Upset plots showing the intersection of different DEU gene sets detected by benchmarked workflows. Only top-10 intersection sets are shown

Figure 4A shows results of DEU gene detection between LP and ML MECs by *DEU-edgeR* and *DEJU-edgeR*. Overall, about 55% of positive discoveries were exclusively reported by *DEJU-edgeR* using the gene-level Simes and *F*-test. Full lists of DEU genes only detected by *DEJU-edgeR* with the gene-level Simes method or *F*-test can be accessed via Supplementary Table S4, Additional File 3.

Of particular interest was the detection of *Fgfr1*, *Myl6*, and *Mvb12a*, whose roles have been reported in signaling pathways crucial for MEC differentiation and development. For instance, Zhao et al. [19] observed the differential expression of spliced variants of *Fgfr1* that govern cell differentiation. Similarly, the expression levels of *Myl6* have been identified as a potential marker for distinguishing epithelial cell populations [20, 21]. Furthermore, *Mvb12a* has been implicated in the regulation of the epidermal growth factor receptor (EGFR) signaling pathway [22]. While alternative splicing events involving these genes have been modestly reported in previous studies, the splicing signals identified in our study warrant further investigation to elucidate their potential roles in regulating MEC differentiation and development.

Figure 4B highlights the differentially used junctions of *Fgfr1* detected by *DEJU-edgeR* (adjusted *P* value = 0.018) but not by *DEU-edgeR* (adjusted *P* value = 1). See Supplementary Fig. S9, Additional File 1 for more illustrative examples of DEU genes exclusively detected by *DEJU-edgeR* with the gene-level Simes method or *F*-test. Figure S4C illustrates the intersections among DEU gene sets detected by different methods. Overall, *DEJU-edgeR/limma* identified more DS discoveries than *DEU-edgeR/limma* (Supplementary Table S5, Additional File 1), underscoring the importance of junction counts in enhancing DS detection. Among all DEU/DEJU methods implemented in *edgeR* and *limma*, *DEJU-edgeR* with the gene-level *F*-test detected the highest number of DEU genes. Only a small subset of DEU genes was detected by all benchmarked methods, with a substantial number of unique DEU genes detected by *DEXSeq* and *JunctionSeq*. Full intersection sets are shown in Supplementary Fig. S10, Additional File 1. More examples of DEU genes detected by *DEJU-limma*, *DEXSeq*, and *JunctionSeq* are further visualized in Supplementary Figs. S11–S13, Additional File 1.

## Methods

### Reference genome

We retrieved the primary genome sequence FASTA file and the comprehensive gene annotation GTF file for the GRCm39 mouse genome release M32 from the GENCODE database (https://www.gencodegenes.org). The GTF file was used to generate comprehensive annotations for genomic features, including flattened exons and splice junctions. To create flattened and merged exon annotation, the *flattenGTF* function of the *Rsubread* package [23] was employed to extract exonic regions from the GTF file and merge overlapping exons attributed to the same gene. In addition, a splice junction annotation file, referred to hereafter as junction database, was generated from annotated exonic regions at the transcript level. Two resultant annotation files were saved in a standard annotation format (SAF). See Supplementary Methods section 3.1, Additional File 1 for more details.

### Simulated datasets

#### Customized transcriptome

To create a customized transcriptome for simulation studies, a subset of 5000 multi-exon protein-coding genes with two transcripts per gene were randomly generated. The annotated exonic regions of the selected genes were extracted and the overlapping exons attributed to the same gene were flattened and merged. Transcripts from the resulting transcriptome were manually modified to mimic the common alternative splicing patterns: ES, MXE, ASS, and IR in equal proportions. These simulation strategies were then repeated using five transcripts per gene to evaluate the performance and robustness of each workflow in a more complex, multi-transcript setting. See Supplementary Methods section 3.2, Additional File 1 for more details.

#### Simulation of RNA-seq sequence reads

The *simReads* function in the Bioconductor package *Rsubread* was used to simulate 75 base pair (bp) long paired-end RNA-seq sequence reads based on the customized transcriptome and the outputs were stored in FASTQ format. The baseline expression levels of transcripts were simulated following the Zipf’s law [24]. Biological variations between replicates within each group were simulated following a gamma distribution. Different library sizes were considered in simulation. In a balanced scenario, the library size was set at 50 million reads across all the samples, whereas in an unbalanced case they were alternating between 25 million and 100 million reads over all samples. Two groups of sample were simulated with different number of biological replicates per group (3, 5, or 10) under different scenarios. To simulate genuine differential splicing events, 1000 out of the total 5000 genes were randomly selected and the four common alternative splicing patterns (ES, MXE, ASS, and IR) were simulated for those 1000 selected genes in equal proportions. For each of those 1000 genes, the baseline abundance of one isoform was increased in the first group and the other isoform in the second group multiplicatively by a fold-change of 3. Null simulations in which underlying transcript abundances are consistent across replicates of both groups were also generated to assess type I error rate control. For each combination of library size and number of biological replicates per group, the simulation was run 20 times, and the results represent the average across those 20 runs. In the five-transcript-per-gene scenario, we evaluated setups with 3 or 10 replicates per group across five independent simulation runs. See Supplementary Methods section 3.2, Additional File 1 for more details.

### Methods benchmarking and evaluation

We assessed the performance of the DEJU workflow and other existing DEU/DEJU approaches in terms of their statistical power in detecting DEU genes, the control of FDR, as well as their computational resource usage. In particular, the *diffSpliceDGE* function of *edgeR* and the *diffSplice* function of *limma* were used for downstream statistical testing as part of the DEJU workflow, denoted as *DEJU-edgeR* and *DEJU-limma*, respectively. These two functions were also used in the standard DEU workflows, denoted as *DEU-edgeR* and *DEU-limma*, where the RNA sequencing reads were assigned to exons only using the default settings of *featureCounts* in *Rsubread*. Other workflows being considered in this benchmarking study include *DEXSeq* and *JunctionSeq*. We used the *featureCounts* function in *subread* package to preprocess exon counts for DEXSeq, which was followed by the DEU analysis using the *DEXSeq* function in *DEXSeq* package with default settings. FDR at the gene-level was controlled using the *perGeneQValue* function of *DEXSeq* package. On the other hand, we used *QoRTs* [25] software package with -–runFunctions writeKnownSplices,writeNovelSplices,writeSpliceExon options to generate exon and splice junction counts which were in turn used as input of *JunctionSeq* to test for differentials of exon and splice junctions using default settings.

To evaluate the robustness and accuracy in the DEU analysis of the compared tools, different simulation settings were applied, considering the number of samples per group and balanced/unbalanced library size scenarios. The maximum memory usage and the computing speed of the DEU/DEJU analysis were also recorded for each compared workflow.

Full details of the simulation and benchmarking procedures are included in Supplementary Methods Section 3.2, Additional File 1.

### RNA-seq profiles of mouse mammary epithelium

All benchmarked workflows in the simulation study were applied to an Illumina short-read paired-end RNA-seq experiment exploring the adult mouse epithelial mammary gland [16]. Three biological replicate samples were obtained for each of the two cell populations of interest, including LP and ML. The raw sequencing data in FASTQ format of all six samples were downloaded from the NCBI Gene Expression Omnibus (GEO) series GSE227748. Paired-end reads were first mapped to the mouse reference genome using the *STAR* aligner with the 2-pass mode. The same reference genome set was used for the DEU/DEJU analysis of the case study. To improve the overall mapping accuracy, non-canonical junctions were excluded using an extra option -–outFilterIntronMotifs RemoveNoncanonical in the second pass. Genes without valid gene symbols were also removed from the analysis. Filtering, normalization, and downstream statistical testing were conducted using the same methods for each workflow as described in the previous simulation study. Full details of the case-study analyses are included in Supplementary Methods section 3.3, Additional File 1.

## Discussion

Here, we present a DEJU analysis strategy - an improved version of the existing DEU method implemented in *edgeR* and *limma*. It enables fast and accurate identification of genes exhibiting DS events between experimental conditions by testing for differential exon and splice junction usage. The proposed *featureCounts* read-counting strategy in the DEJU workflow addresses the double-counting issue commonly seen in standard DEU analyses. Double-counting occurs when a single read is assigned to multiple features, inflating exon counts and introducing overdispersion in the modeling process. By separately quantifying exon–exon junction reads and internal exon reads, this approach ensures that library sizes accurately reflect the true number of sequenced reads, leading to improved dispersion estimates and better model fitting. Our DEJU workflow also enhances junction read mapping accuracy by utilizing the two-pass mapping strategy implemented in *STAR*. By offering flexible parameter settings for junction filtering and leveraging an indexed genome with annotated junction information, *STAR* is a consistently accurate and computationally effective performer in junction read mapping, as reported in several benchmark studies [26–28]. We also generate the junction database based on genomic annotation to facilitate junction annotation and improve the accuracy of junction read counts.

Previous benchmark studies have shown that *DEU-edgeR* and *DEXSeq* effectively control FDR at 0.05 across various RNA-seq studies [14, 29, 30]. In this study, we benchmark the newly developed DEJU method in *edgeR* and *limma* against existing tools using a simplified transcriptome designed to simulate common splicing events. Unlike traditional DEU methods that rely soly on exon counts, DEJU incorporates exon-junction reads, enhancing detecting power in ASS and RI events, while maintaining high sensitivity, computational efficiency, and effective FDR control for any combinations of sample sizes and library balance. The DEJU approach enhances detection of ASS by leveraging differential signals from exon-junction usage, particularly in cases where read counts of truncated exons with few base-pair overhangs at splice sites do not contribute significantly to overall differentials in flattened exons. Besides, RI, which is non-detectable by existing DEU methods due to the lack of intron reads, can be detected by the DEJU method owing to exon-junction reads. *DEXSeq* and *JunctionSeq* require substantially more computational time and resources scaled with increasing sample sizes. *DEXSeq* was reported to encounter indefinite run-time issue when handling a large number of replicate samples [30]. Besides, *JunctionSeq* is no longer supported by R/Bioconductor, and its latest version conflicts with *DESeq2* v1.15+, which is externally used by *JunctionSeq* to model count dispersion. Regarding statistical testing, *DEJU-edgeR/limma* provides both exon- and gene-level adjusted *P* values using Simes and F-test. Simes test is recommended over the *F*-test when only a subset of exons or junctions within a gene exhibits differential usage. However, since multiple DS events can occur within the same gene in a complex manner, the *F*-test may be more reliable for capturing overall gene-level splicing changes. *DEXSeq* and *JunctionSeq* only provide a single gene-level test. Besides, exon-level tests help pinpoint specific gene regions undergoing DS. By incorporating multiple significance tests, our DEJU method provides more comprehensive results of DS detection compared to *DEXSeq* and *JunctionSeq*.

The DEJU workflow is an efficient and powerful way for detecting DEU genes, as demonstrated through both simulation studies and real RNA-seq datasets. DEJU captures a more comprehensive landscape of DS events, including exon truncation/extension with alternative splice sites, nested exon skipping, intron retention, or novel isoforms driven by distal alternative promoters (Fig. 4B, Supplementary Fig. S9, Additional File 1). It also demonstrates optimal performance when applied to datasets with large numbers of replicates, as shown in our simulation studies. In the multi-transcript simulation studies, where low-abundance isoforms involved in DS events are inherently more challenging to detect, the DEJU workflow maintains robust performance, though sensitivity is somewhat reduced for ASS and IR events. Importantly, increasing the number of replicates leads to substantial gains in detection power. This scalability makes the DEJU workflow particularly well-suited for large-scale cancer cohorts, where larger sample sizes can enhance the identification of biologically meaningful splicing events. For instance, the Cancer Genome Atlas (TCGA), which includes RNA-seq data from hundreds of tumor and normal samples, presents a valuable opportunity to apply DEJU methods. These applications could reveal novel AS events in cancer-related genes, offering potential targets for further biological and clinical research. In addition, our case study of the mouse mammary gland further illustrates the advantages of the DEJU method, revealing DEU genes that were not previously identified. Notably, these newly detected genes are supported by biologically meaningful interpretations.

Other popular DS tools, notably *rMATS* [31], *MAJIQ* [32], *LeafCutter* [33], *SUPPA*/*SUPPA2* [34], and *junctionCounts* [35], detect DS genes from differential splicing event (DSE) analysis, which also leverages exon-junction reads. Different from DEJU, DSE only consider exon-junction reads at the event level to calculate percent spliced-in values [36]. Previous studies have benchmarked DSE tools against the existing DEU tools [14, 29, 30]. DSE tools, *MAJIQ* and *rMATS*, achieve relatively higher number of validated DS genes, more effective FDR control, and higher precision. However, some of DSE tools, such as *junctionCounts*, *MAJIQ*, or *SUPPA/SUPPA2*, do not report adjusted *P* values, which are essential for assessing the confidence of DS detections and FDR control for top-ranked DS genes. Regarding computational efficiency, *DEU-edgeR/limma* outperforms DSE tools. From a usability perspective, existing DEU tools offer greater flexibility for complex experimental designs, pairwise comparisons, and confounder adjustments, whereas DSE tools are typically limited to two-group comparisons [14]. Although *DEJU-edgeR/limma* improves power and FDR control over existing DEU tools, further benchmarking against DSE tools is neccessary, yet beyond the scope of this study.

While our DEJU method shows strong promise and broad applicability for future DS studies, our study has several limitations. First, our simulations were based on a simplified transcriptome with only two or five transcripts per gene. Additional complexities commonly found in real biological datasets, such as outlier samples, technical biases, and batch effects, were not incorporated into the simulation design. Accurately modeling these factors would require substantially more sophisticated strategies, which are beyond the scope of the current study. Second, the DEJU workflow is not currently applicable to single-cell RNA-seq data. In fact, most of the methods benchmarked in this study are also not suitable for single-cell settings. This limitation arises because the majority of single-cell RNA-seq data are generated using 3′ polyA capture protocols (e.g., 10× Genomics), where sequencing reads typically cover only 90–100 base pairs from the 3′ end of each transcript. Such limited coverage, combined with the inherent sparsity of single-cell data, makes it challenging to perform DEU analysis at the resolution achievable with bulk RNA-seq.

## Conclusions

By incorporating exon–exon junction reads, our DEJU workflow demonstrates strong performance in FDR control, statistical power, computational efficiency, and flexibility in detecting a broad range of AS events, making it the most suitable candidate for DEJU analysis in RNA-seq data. In practical applications, our DEJU method effectively handles splicing alterations involving multiple known and novel transcripts, while supporting complex experimental designs. The effectiveness of the DEJU approach increases with sample size, making it particularly advantageous in large-scale studies. However, it is not recommended for non-model organisms with the incomplete reference genome and does not provide transcript-level abundance estimates.

## Additional file

Additional file 1: Supplementary Materials for Incorporating exon–exon junction reads enhances differential splicing detection. It includes Supplementary Figures S1–13, Supplementary Tables S2-S3, S5, and Supplementary Methods.

Additional file 2: Supplementary Table S1. Summary of the average and standard error of FDR and statistical power in the DEU detection across benchmarked methods.

Additional file 3: Supplementary Table S4. Lists of DEU genes of LP against ML samples exclusively detected by *DEJU-edgeR* using the gene-level Simes method and *F*-test. The simulation and analysis code to reproduce the results shown in this article are available from https://github.com/TamPham271299/DEJU.

## Data Availability

All the data are publicly available as described in the Methods section.

## Abbreviations

RNA-seq: RNA sequencing
DEU: Differential exon usage
DEJU: Differential exon-junction usage
AS: Alternative splicing
DS: Differential splicing
FDR: False discovery rate
MEC: Mammary epithelial cell
TMM: Trimmed mean of M values
ES: Exon skipping
MXE: Mutually exclusive exon
ASS: Alternative 3′/5′ splice site
IR: Intron retention
LP: Luminal progenitor
ML: Mature luminal
EGFR: Epidermal growth factor receptor
SAF: Standard annotation format
DSE: Differential splicing event
